# Supplementary material for: Multi-Omics Insights into Rumen Microbiota and Metabolite Interactions Regulating Milk Fat Synthesis in Buffaloes
Source: Animals (Basel). 2025 Jan 17;15(2):248. doi: 10.3390/ani15020248 (PMC11758634; doi:10.3390/ani15020248)
Supplement: Supplementary file 1 [file animals-15-00248-s001.zip › Table S1.pdf]

Table S1. Feed ingredients of the buffaloes.

Ingredients	Content%
Concentrate mixture	35
Corn silage	48
Rice straw	5
Elephant grass	12
