# Supplementary material for: Multi-Omics Insights into Rumen Microbiota and Metabolite Interactions Regulating Milk Fat Synthesis in Buffaloes
Source: Animals (Basel). 2025 Jan 17;15(2):248. doi: 10.3390/ani15020248 (PMC11758634; doi:10.3390/ani15020248)
Supplement: Supplementary file 1 [file animals-15-00248-s001.zip › Table S2.pdf]

Table S2. Concentrate mixture of the buffaloes.

Ingredients	Content%
Corn	45
Soybean meal	20
Wheat bran	25
Rapeseed meal	5
Premix	5

Premix contained (per kg) the following: Fe, 3,900 mg; Cu, 900 mg; Zn, 2,500 mg; Mn, 1200 mg; Se, 10mg; Ca, 12%; P, 6%; Nacl, 5%; vitamin A, 1,500 kIU; vitamin D3, 500 kIU; and vitamin E, 5,500 IU.
