# Supplementary material for: Multi-Omics Insights into Rumen Microbiota and Metabolite Interactions Regulating Milk Fat Synthesis in Buffaloes
Source: Animals (Basel). 2025 Jan 17;15(2):248. doi: 10.3390/ani15020248 (PMC11758634; doi:10.3390/ani15020248)
Supplement: Supplementary file 1 [file animals-15-00248-s001.zip › Table S3.pdf]

**Table S3.** The concentration of VFAs in rumen fluid of HF and LF buffaloes.

| Items | Mean±SEM | | *P* |
|---|---|---|---|
| | HF (n=10) | LF (n=10) | |
| Acetate(mmol/L) | 37.54±1.33 | 30.46±1.71 | 0.004 |
| Propionate(mmol/L) | 7.14±0.32 | 7.03±0.50 | 0.85 |
| Isobutyrate(mmol/L) | 0.51±0.07 | 0.45±0.02 | 0.45 |
| Butyrate(mmol/L) | 4.01±0.42 | 3.40±0.22 | 0.21 |
| Isovalerate(mmol/L) | 0.37±0.06 | 0.31±0.02 | 0.36 |
| Valerate(mmol/L) | 0.39±0.08 | 0.37±0.03 | 0.80 |
| Total volatile fatty acids (TVFAs, mmol/L) | 49.85±1.93 | 42.15±2.00 | 0.012 |
