# Supplementary material for: Multi-Omics Insights into Rumen Microbiota and Metabolite Interactions Regulating Milk Fat Synthesis in Buffaloes
Source: Animals (Basel). 2025 Jan 17;15(2):248. doi: 10.3390/ani15020248 (PMC11758634; doi:10.3390/ani15020248)
Supplement: Supplementary file 1 [file animals-15-00248-s001.zip › Table S4.pdf]

**Table S4.** Summary of sequence data generated from rumen samples of HF and LF buffaloes.

| Sample\Info | Seq_num | Base_num | Mean_length | Min_length | Max_length |
|---|---|---|---|---|---|
| HF1 | 43685 | 18326043 | 420 | 217 | 455 |
| HF2 | 51513 | 21493644 | 417 | 235 | 511 |
| HF3 | 39286 | 16391848 | 417 | 204 | 488 |
| HF4 | 47739 | 19818647 | 415 | 249 | 435 |
| HF5 | 45262 | 19001308 | 420 | 242 | 437 |
| HF6 | 48268 | 20217445 | 419 | 317 | 491 |
| HF7 | 47945 | 19963580 | 416 | 215 | 455 |
| HF8 | 47704 | 19999156 | 419 | 259 | 439 |
| HF9 | 62911 | 26272449 | 418 | 245 | 446 |
| HF10 | 46188 | 19293334 | 418 | 232 | 472 |
| LF1 | 53036 | 22245650 | 419 | 214 | 451 |
| LF2 | 43617 | 18201990 | 417 | 203 | 531 |
| LF3 | 45308 | 18971779 | 419 | 319 | 511 |
| LF4 | 44878 | 18708230 | 417 | 252 | 468 |
| LF5 | 66230 | 27719409 | 419 | 213 | 442 |
| LF6 | 48049 | 20090930 | 418 | 252 | 435 |
| LF7 | 45321 | 18991581 | 419 | 219 | 445 |
| LF8 | 52514 | 21891955 | 417 | 262 | 486 |
| LF9 | 43239 | 17937210 | 415 | 208 | 435 |
| LF10 | 38120 | 15936548 | 418 | 247 | 516 |
| Total | 960813 | 401472736 | 8357 | 4804 | 9349 |
| Mean | 48041 | 20073637 | 418 | 240 | 467 |
| SEM | 1523 | 638311 | 0 | 7 | 7 |
