# Supplementary material for: Multi-Omics Insights into Rumen Microbiota and Metabolite Interactions Regulating Milk Fat Synthesis in Buffaloes
Source: Animals (Basel). 2025 Jan 17;15(2):248. doi: 10.3390/ani15020248 (PMC11758634; doi:10.3390/ani15020248)
Supplement: Supplementary file 1 [file animals-15-00248-s001.zip › Table S5.pdf]

Table S5. Summary of sequence data generated from rumen samples of HF and LF buffaloes.

Sample\Info	Seq_num	Base_num	Mean_length	Min_length	Max_length
HF1	74251	14626509	197	140	515
HF2	54657	13567264	248	141	368
HF3	56851	13113948	231	141	491
HF4	74247	15658869	211	140	338
HF5	72714	14371334	198	140	344
HF6	55225	13922904	252	141	345
HF7	50754	12461149	246	140	369
HF8	71274	14922230	209	140	340
HF9	51501	12677930	246	141	344
HF10	58934	13868848	235	140	527
LF1	70376	15092758	214	140	517
LF2	71438	15788239	221	141	525
LF3	45120	10645410	236	140	490
LF4	34117	8949951	262	141	492
LF5	47007	12117409	258	140	493
LF6	59021	13434392	228	140	437
LF7	63990	13662136	214	140	474
LF8	62233	14308634	230	140	503
LF9	61438	13109770	213	140	499
LF10	74815	15824480	212	140	522
Total	1209963	272124164	4560	2806	8933
Mean	60498	13606208	228	140	447
SEM	2540	382119	4	0	17
