# Supplementary figures and images for: Multi-Omics Insights into Rumen Microbiota and Metabolite Interactions Regulating Milk Fat Synthesis in Buffaloes
Source: Animals (Basel). 2025 Jan 17;15(2):248. doi: 10.3390/ani15020248 (PMC11758634; doi:10.3390/ani15020248)

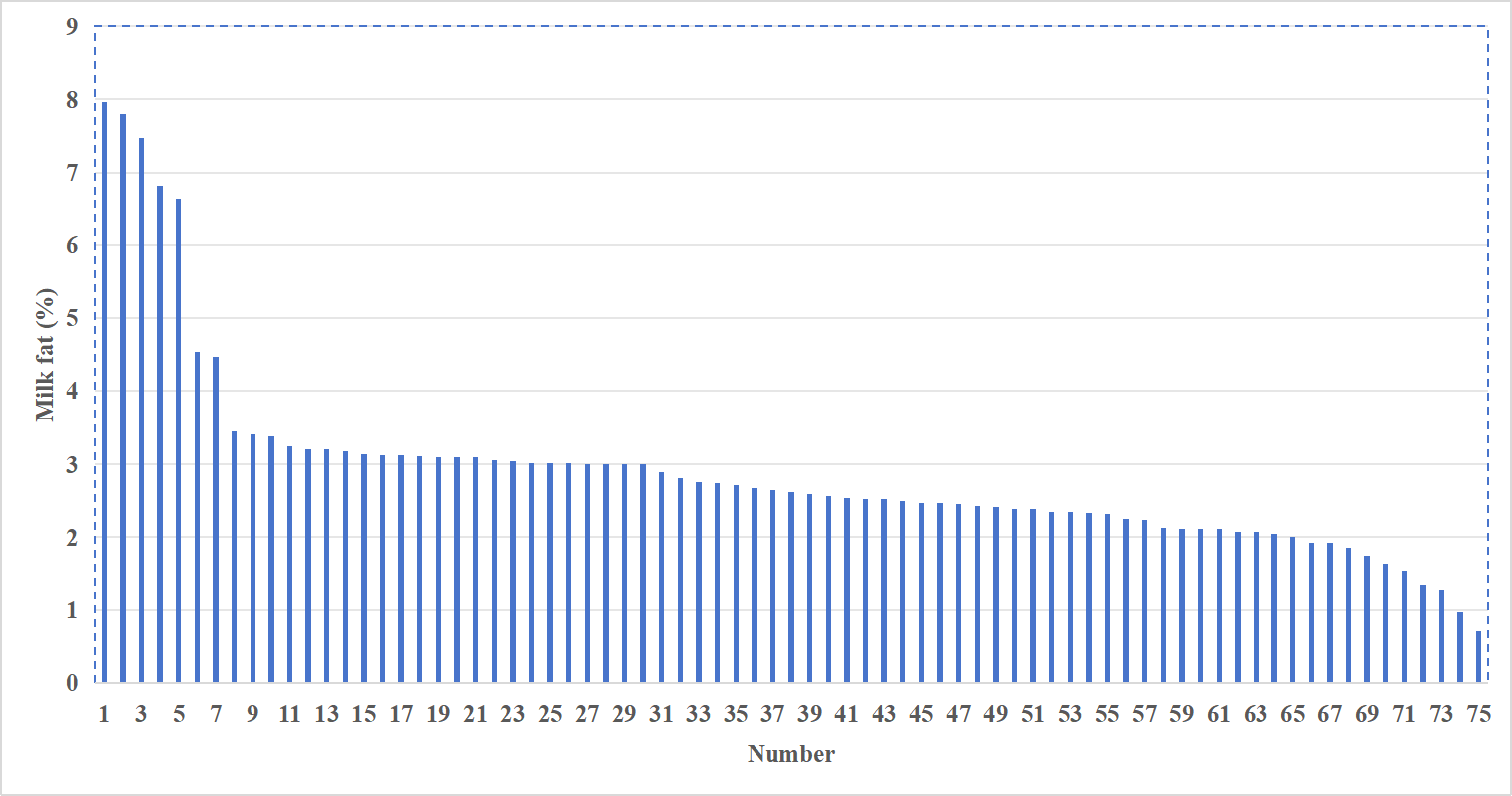

Supplement: Supplementary file 1 [file animals-15-00248-s001.zip › Figure S1.png]

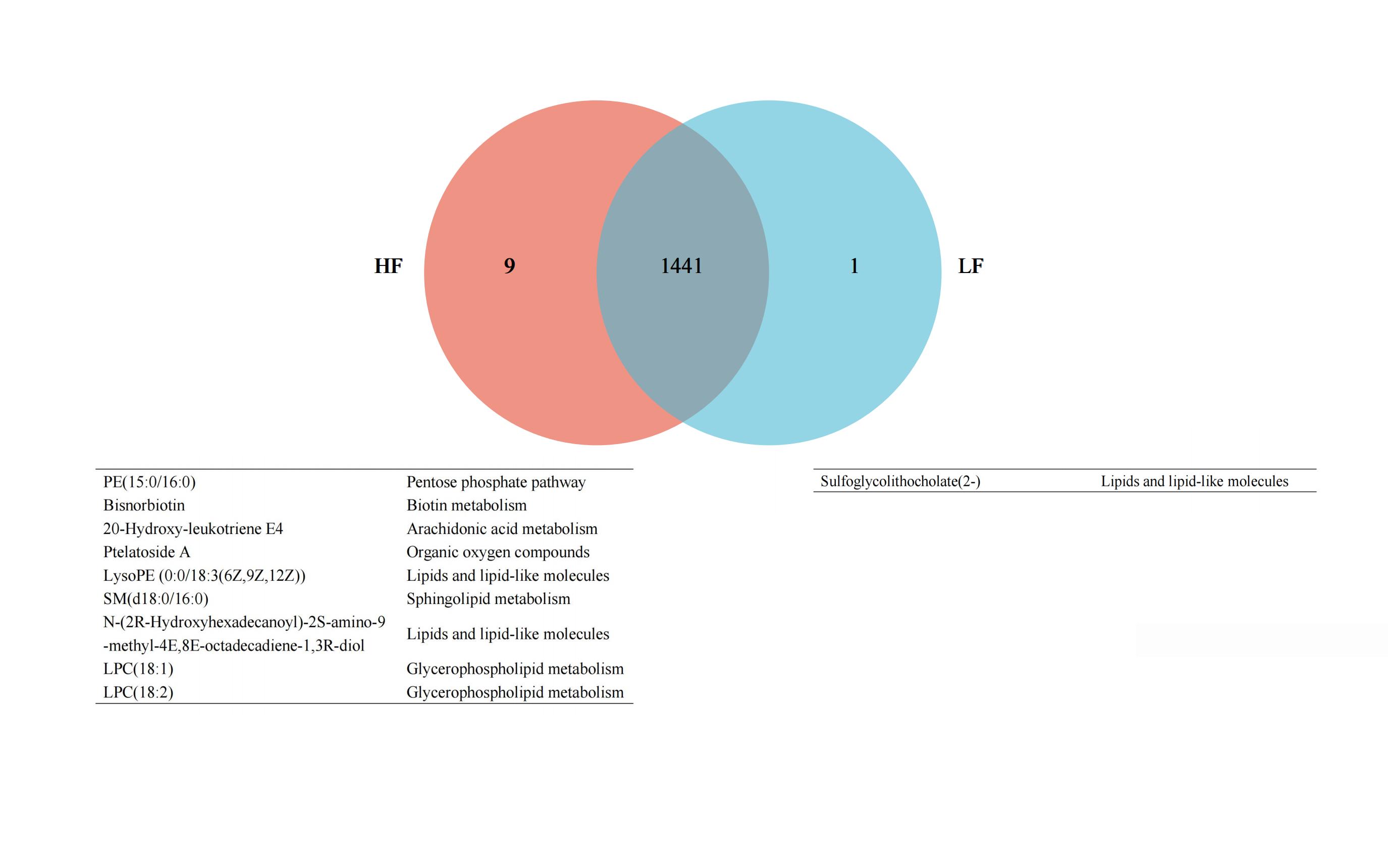

Supplement: Supplementary file 1 [file animals-15-00248-s001.zip › Figure S2.jpg]

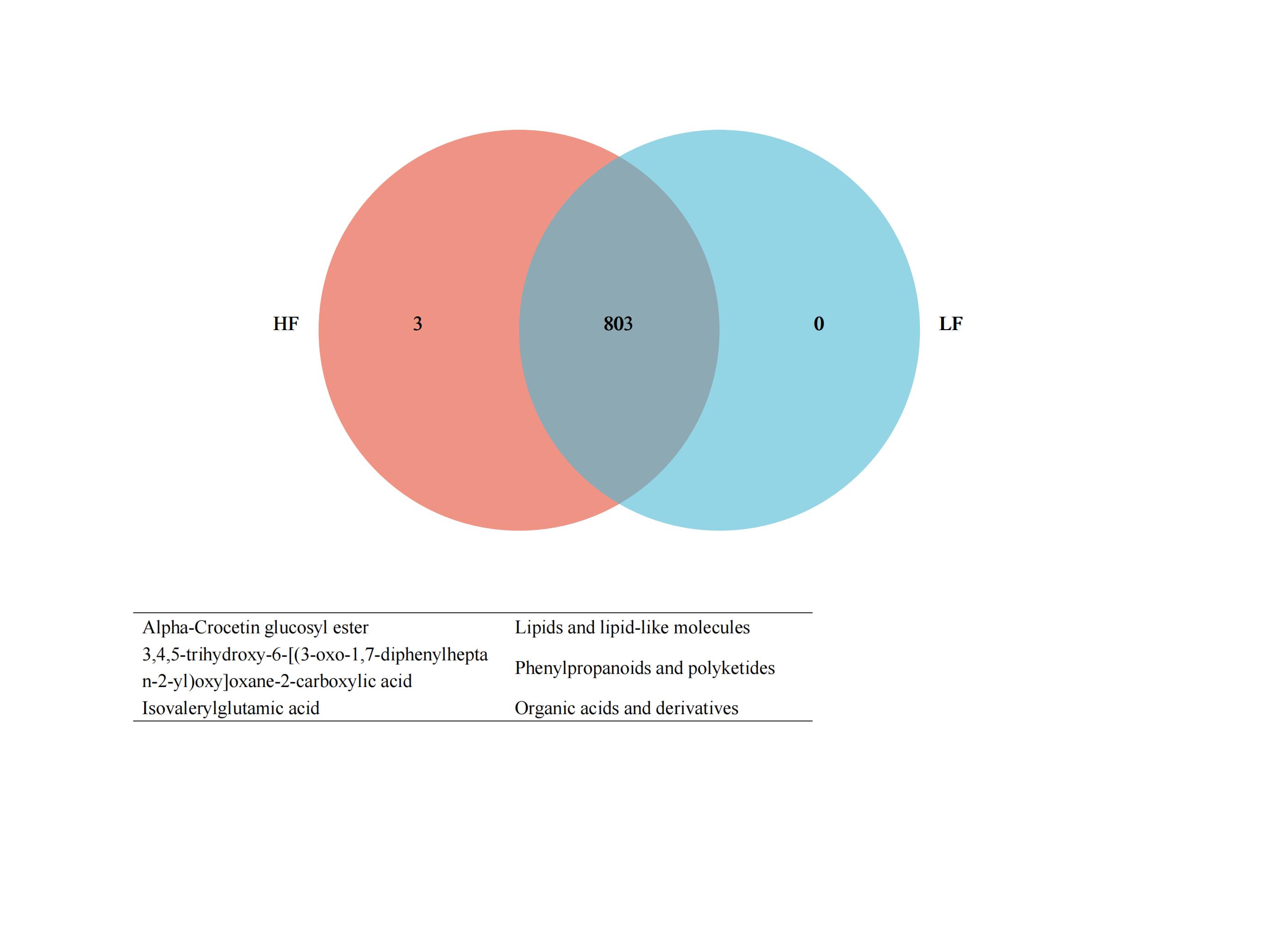

Supplement: Supplementary file 1 [file animals-15-00248-s001.zip › Figure S3.jpg]

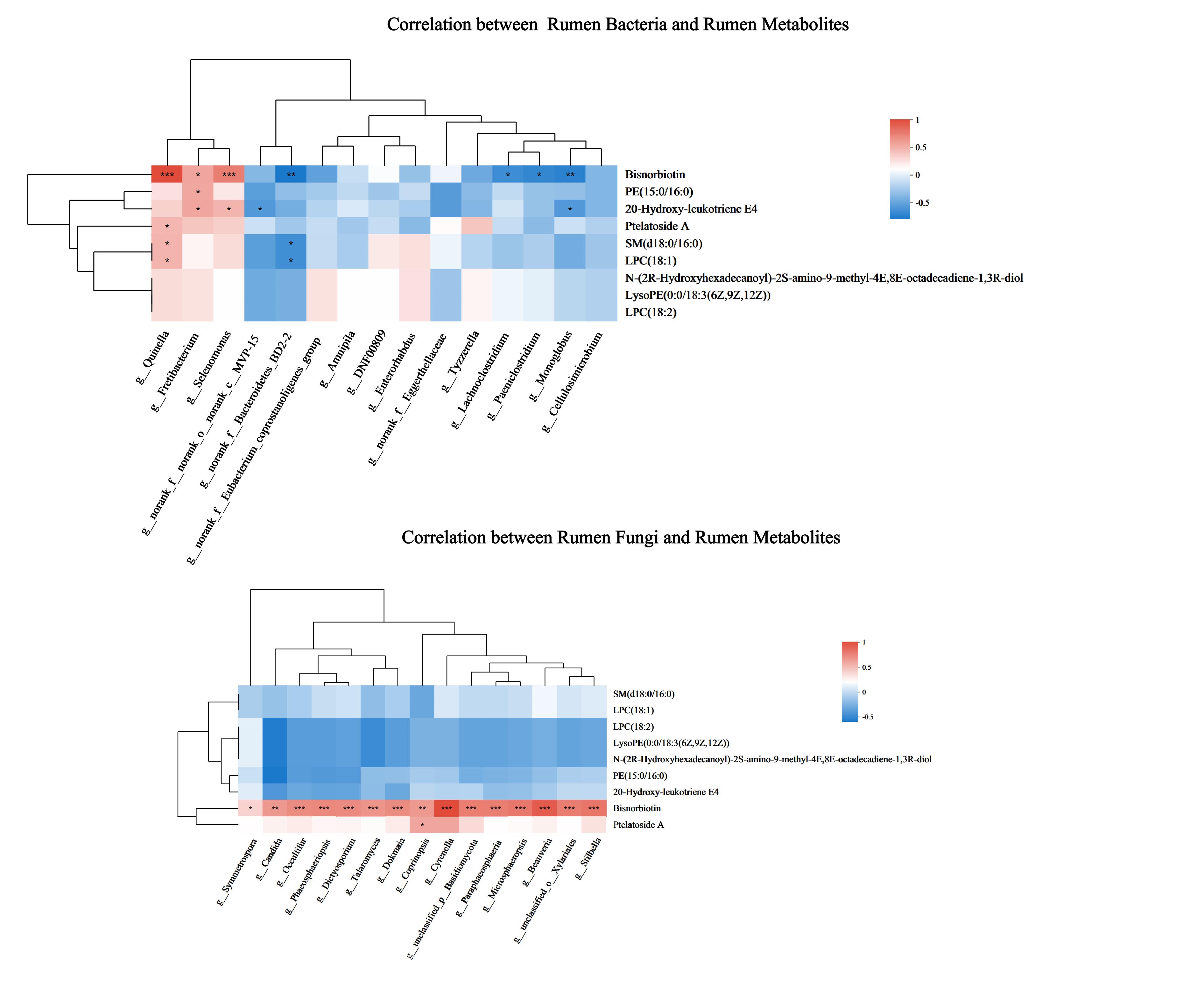

Supplement: Supplementary file 1 [file animals-15-00248-s001.zip › Figure S4.jpg]

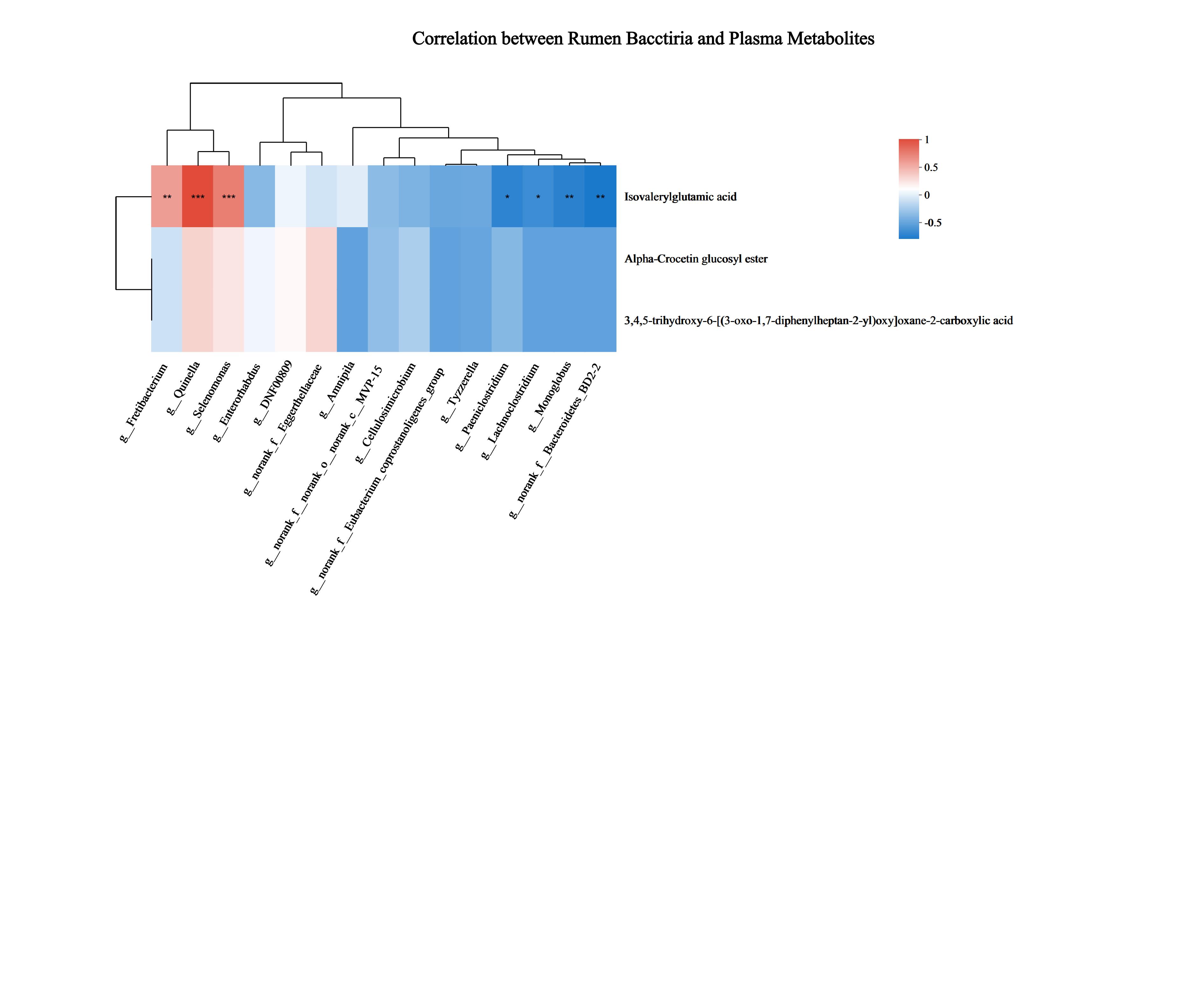

Supplement: Supplementary file 1 [file animals-15-00248-s001.zip › Figure S5(A).jpg]

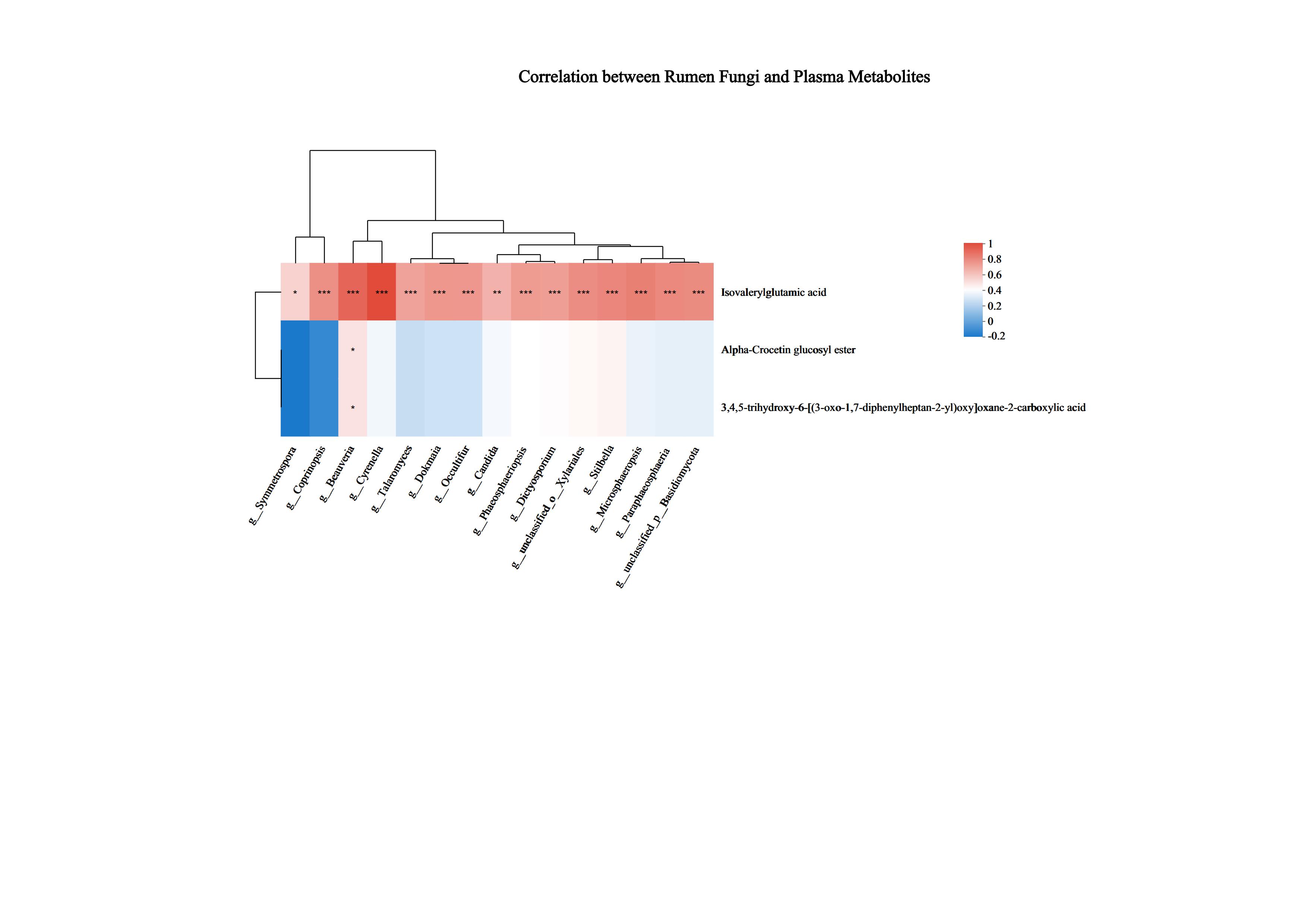

Supplement: Supplementary file 1 [file animals-15-00248-s001.zip › Figure S5(B).jpg]
